# Bilateral proptosis—an unusual presentation of extensive allergic fungal sinusitis

**DOI:** 10.1093/jscr/rjaa564

**Published:** 2022-10-21

**Authors:** Ali Almomen, Omar Al-enzi, Reem Alshammari, Fadel Molani, Abdullah A Alshakhs, Mohammed A Al Ameer, Raghad A Alarim, Mohammed A Alfalah, Jenan A Marhoon

**Affiliations:** King Fahad Specialist Hospital, Dammam, Kingdom of Saudi Arabia; King Fahad Specialist Hospital, Dammam, Kingdom of Saudi Arabia; College of Medicine, Northern Borders University, Arar, Saudi Arabia; King Fahad Specialist Hospital, Dammam, Kingdom of Saudi Arabia; College of Medicine, King Faisal University, AlAhsa, Kingdom of Saudi Arabia; College of Medicine, King Faisal University, AlAhsa, Kingdom of Saudi Arabia; College of Medicine, King Khalid University, Abha, Kingdom of Saudi Arabia; College of Medicine, King Faisal University, AlAhsa, Kingdom of Saudi Arabia; College of Medicine, Jordan University of Science and Technology, Irbid, Jordan

## Abstract

Allergic fungal rhinosinusitis (AFRS) is counted as the most common form of fungal sinusitis. It is mainly due to the hypersensitivity reaction to fungal infection. Usually, the patients are atopic or immunocompetent. These patients are usually suffering from signs and symptoms of rhinosinusitis. The expanding mass in the disease leads to bony remodeling and the involvement of adjacent structures. When allergic mucin involves the orbit, many complications may occur. This includes diplopia, telecanthus, unilateral proptosis, malar flattening, epiphora, Asthenopia and even visual loss. The diagnosing of AFRS initially requires radiographic imaging, but to confirm the diagnosis, histopathological examination is needed. The treatment of AFRS should be combined with surgical and medical therapy. This case report demonstrates a unique and rare presentation of the non-invasive AFRS with bilateral proptosis which had dramatic improvement and resolution after we managed it with endoscopic sinus surgery, steroids and nasal saline irrigation.

## INTRODUCTION

Allergic fungal rhinosinusitis (AFRS) is counted as the most common form of fungal sinusitis. It is mainly due to the hypersensitivity reaction to fungal infection. [1] Usually, the patients are atopic or immunocompetent. The ophthalmic manifestations are common in these patients. [2] This article showed a very rare complication of AFS that present with bilateral proptosis in immunocompetent patients. It was diagnosed and managed at King Fahad Specialist Hospital (KFSHD)—a tertiary care hospital in Al-Dammam, Saudi Arabia.

## CASE REPORT

**Figure 1:**
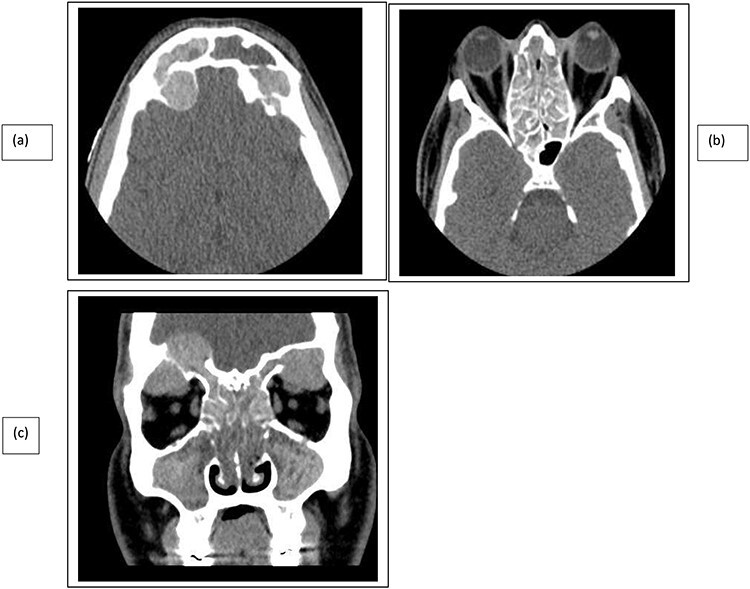
CT paranasal sinuses axial (**a**, **b**) and coronal (**c**) show sinus expansion, bony remodeling and dehiscence with intra-orbital and intracranial extension. Bilateral orbital proptosis.

**Figure 2:**
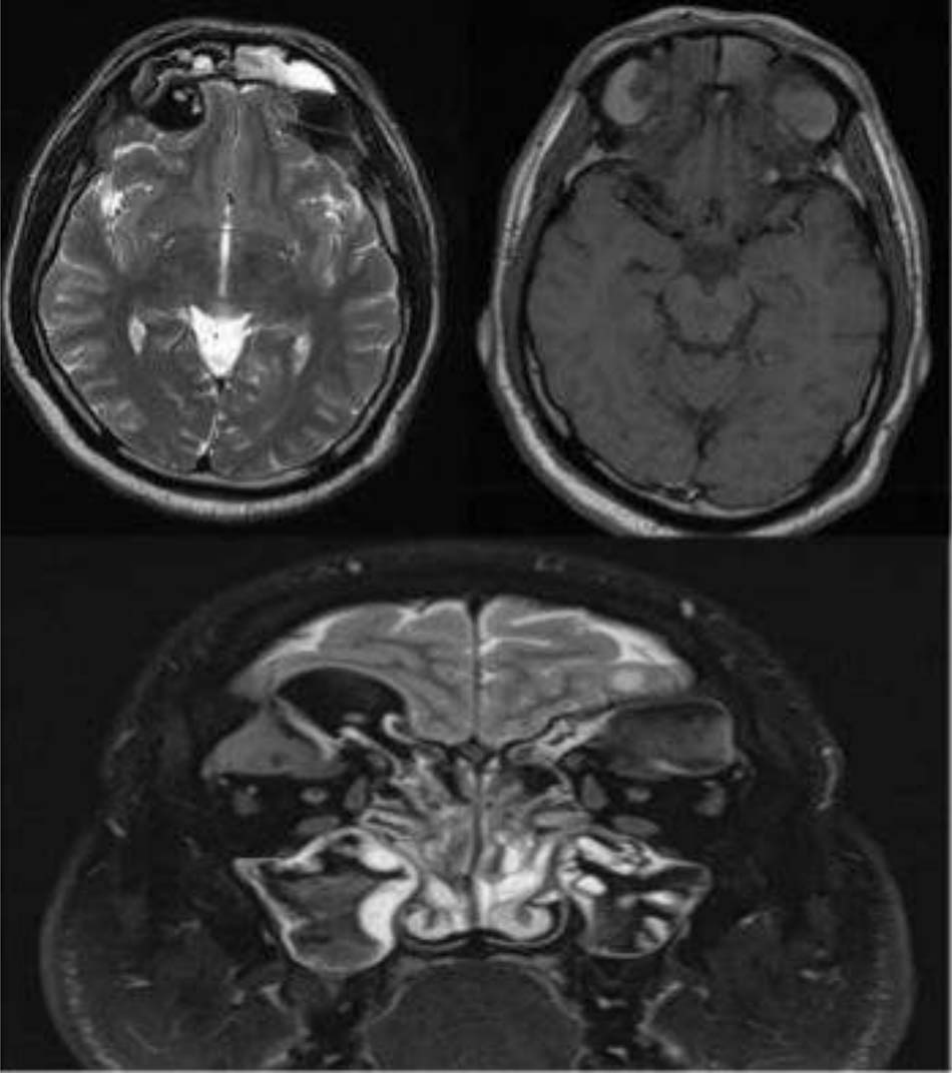
MRI paranasal sinuses axial and coronal T2 and axial T1 The MRI shows the expansion with the remodeling of the sinuses and intra-orbital/intracranial extension. The hyperdense contents show marked low signal intensity on T2 and high T1 signal. No tissue invasion of the orbital or brain parenchyma.

A 35-year-old Saudi male presented to our ENT Clinic with a 1-year history of progressive facial pain, headache, bilateral proptosis, anosmia and nasal obstruction. His past medical history was insignificant. Sinonasal endoscopy revealed multiple bilateral grade IV polyps, occluding the nasal cavities bilaterally. Computed tomography (CT) of the paranasal sinuses showed expansion and remodeling of the frontal sinuses bilaterally (Fig. 1). It is associated with bony dehiscence posterior wall and floor of the sinuses that results in intracranial and intra-orbital extension, respectively. The ethmoid air cells show complete opacification bilaterally with lateral bowing of the lamina papyracea. This along with the frontal sinus orbital extension lead to the clinical presentation of the orbital proptosis. The maxillary antra disease results in remodeling of the stomatal complex and obliteration of the superior two-thirds of the nasal cavities. The contents of the sinuses show mixed iso and hyperdense material. Magnetic resonance imaging (MRI) of the paranasal sinuses demonstrates the orbital and intracranial extension of the disease with no invasion of the adjacent tissues or brain parenchyma (Fig. 2). The hyperdense contents show marked low signal intensity on T2 and high signal intensity on T1.

The patient underwent functional endoscopic sinus surgery. The polyps were removed completely from the nasal cavities, bilateral wide maxillary sinus enterostomies, and thick mucin was aspirated. The anterior and posterior ethmoids sinuses were full of polyps, mucin and fungal mud which was aspirated the lamina papyruses that were dehiscent bilaterally by the polyps causing the proptosis of both eyes. The frontal recesses and sinuses were full of polyps, mucin and fungal mud causing erosion of frontal floors and displacing the orbits, and all were cleaned and drained with the help of navigation assisted surgery. The patient had dramatic improvement after surgery, the rhinosinusitis symptoms and the proptosis resolved, and the paranasal sinuses were clear. The patient was discharged with topical corticosteroid and saline irrigations.

## DISCUSSION

In patients with AFRS, progressive nasal obstruction and multiple nasal polyps are presented with unilateral distribution most of the time. Usually, these patients are suffering from signs and symptoms of rhinosinusitis lasting months or even years, and they may delay visiting the hospital until they develop complete nasal obstruction, visual disturbances or facial disfiguration [2–5]. The accumulation of mucin has relatively unique and predictable characteristics. The expanding mass leads to bony erosion and the involvement of adjacent structures. However, it is important to point out that the disease process itself does not invade tissues. The involvement of adjacent structures is due to the expanding mucin causing ischemia and weakness of bone, making it susceptible to mechanical stress and necrosis, by exerting pressure that compromising the bone blood supply [6, 7].

The direction of the least resistance and the location of the disease will determine the site of expansion. The lamina papyracea is the most common location of bone erosion, and the orbit is the most common location for extra-sinus disease spread [8, 9]. When allergic mucin involved in the orbit, bony remodeling and decalcification will occur, causing the disease to mimic invasion into adjacent structures or cavities which can produce dramatic manifestations such as visual loss or facial disfiguration. There are many complications that may occur due to this process, including, diplopia, telecanthus, unilateral proptosis, malar flattening, epiphora, Asthenopia and even vision loss. The most common among them are unilateral proptosis [1, 2, 9, 10].

Loss of vision can be caused by two mechanisms including, the inflammatory process of adjacent structures that may affect the optic nerve, resulting in optic neuritis or infarction of the optic nerve caused by increased intra-orbital pressure which may lead also to thrombophlebitis within the valveless orbital veins or even occlusion of the central retinal artery which is an ocular emergency [1, 6, 9]. Facial disfiguration is more common in young patients due to the fact that the facial skeleton growth is at its highest rate at this age. During this phase, the growing bony structures become pliable and more susceptible to dysmorphic changes by the external forces; in this case, it is the forces of AFRS expansion, causing bony erosion and skeletal changes of the sinus walls and adjacent cavities such as intracranial or intra-orbital cavities, with the proptosis as the most common result [7, 8].

In advanced cases, the base of the skull may be involved, leading to an intracranial extension which may give rise to frontal lobe compression leading to change in personality and behavior, or even an intracranial abscess [6].

The diagnosing of AFRS initially requires radiographic imaging. Paranasal sinus CT without contrast is the preferred modality. Its findings usually include irregular hyperdense masses involving multiple sinuses. The ethmoid sinus is most commonly affected, followed by the maxillary, frontal and sphenoid sinuses, respectively. MRI is a good alternative, which may reveal enhanced sinus mucosa and masses with markedly decreased intensities resembling air on T2 images. The radiological findings obtained from CT and MRI are characteristic of dense extra-mucosal mucin, but they are not enough to diagnose AFRS. The definitive diagnosis is reached by histopathological examination of mucosa and mucin [7, 9, 10]. Histopathological findings of mucus are the presence of fungal elements, Charcot–Leyden crystals and eosinophils. Cultures of the mucin show dematiaceous fungi species growth most of the time [8, 9].

The treatment of AFRS should be combined with surgical and medical therapy, which consists of sinuses surgical decompression, corticosteroid and nasal saline irrigation. Adjuvant medical therapy aims to decrease inflammation, atopy and antigen exposure, therefore, the immunoreactions. In the absence of this combination, the recurrence rates of AFRS reach up to 100%. [3, 6–9].

## CONCLUSION

This case report demonstrates a unique and rare presentation of the non-invasive AFRS with bilateral proptosis which had dramatic improvement and resolution of the proptosis after we managed it with Endoscopic sinus surgery, steroids and nasal saline irrigation.

## CONFLICT OF INTEREST STATEMENT

The authors declare that there is no conflict of interest regarding the publication of this paper.

## FUNDING

There is no financial support and sponsorship.

## CONSENT

Written informed consent was obtained from the parents for publication of this case report on behalf of the patient.
